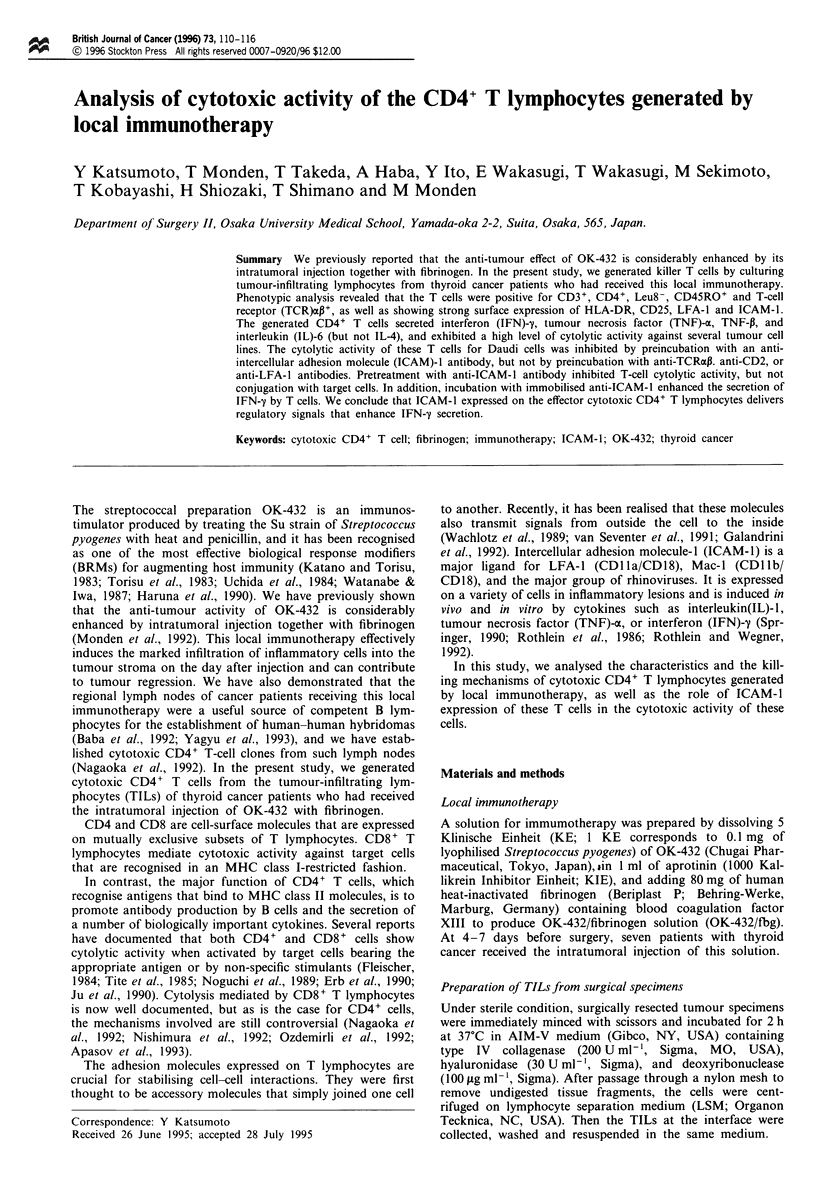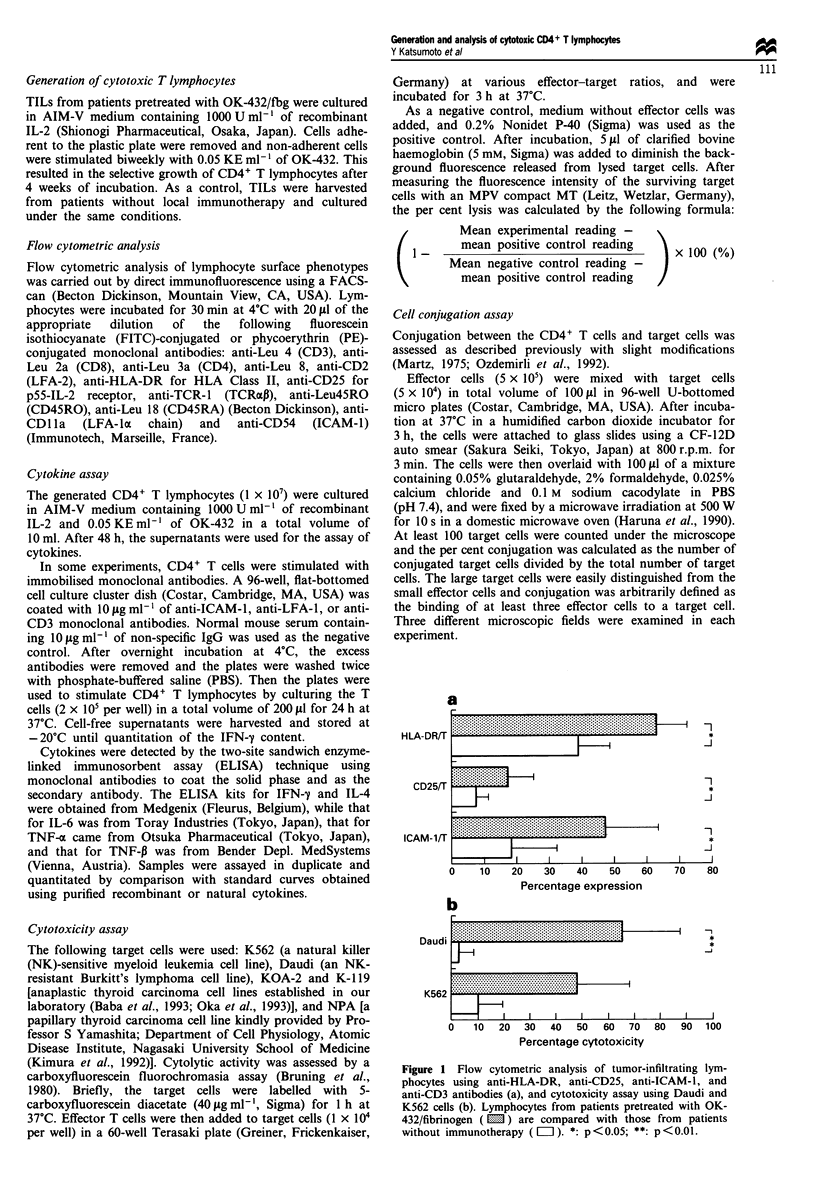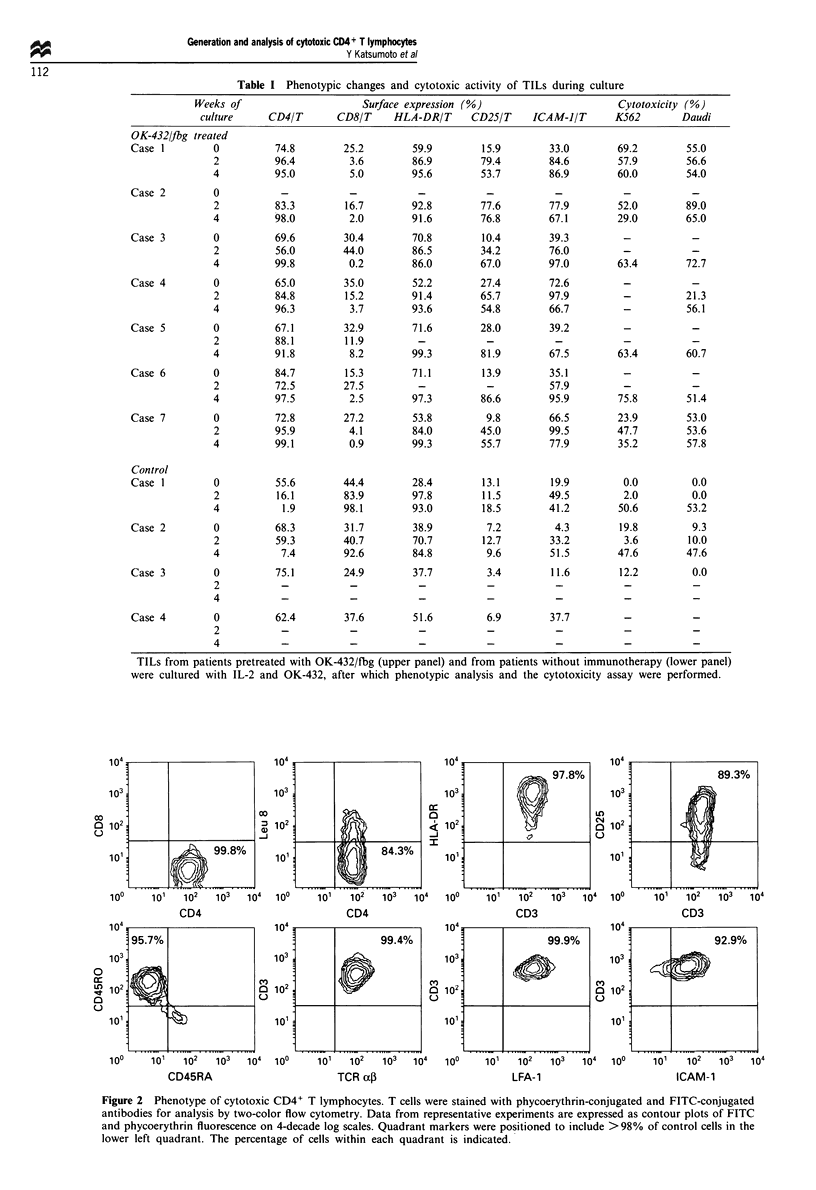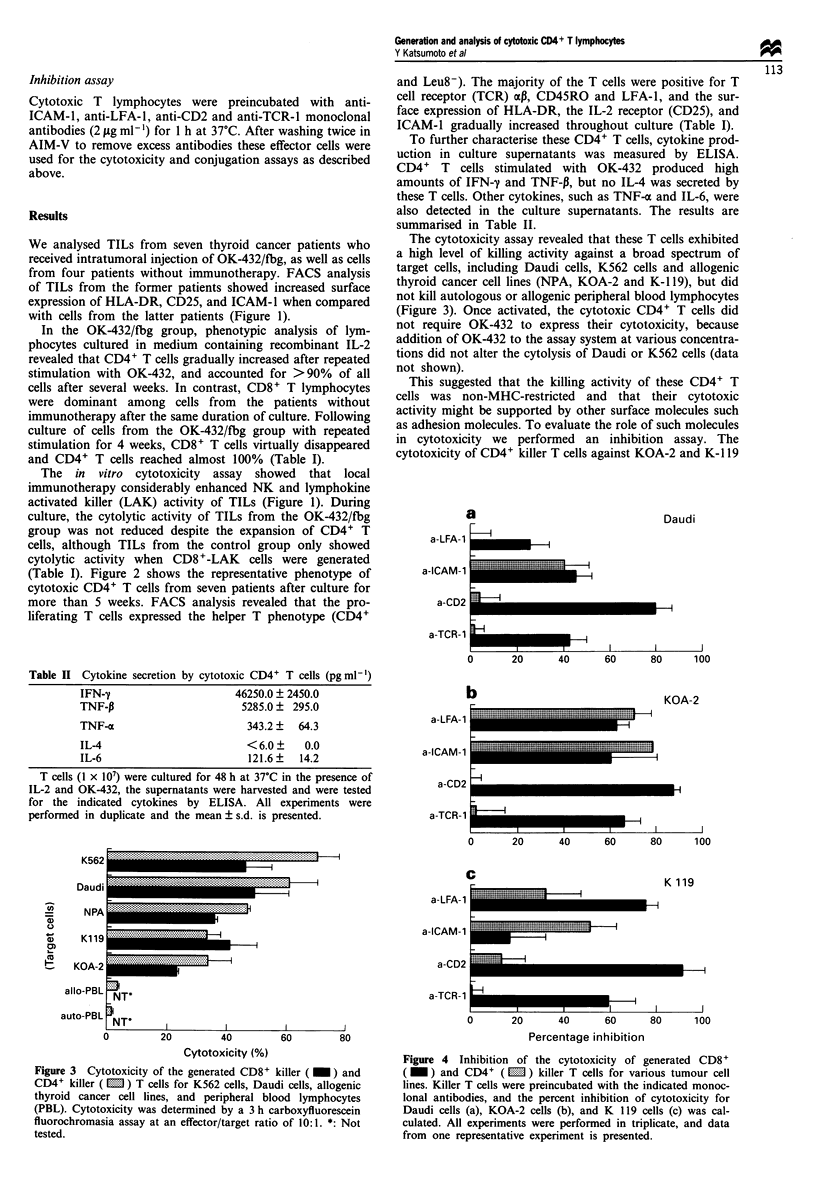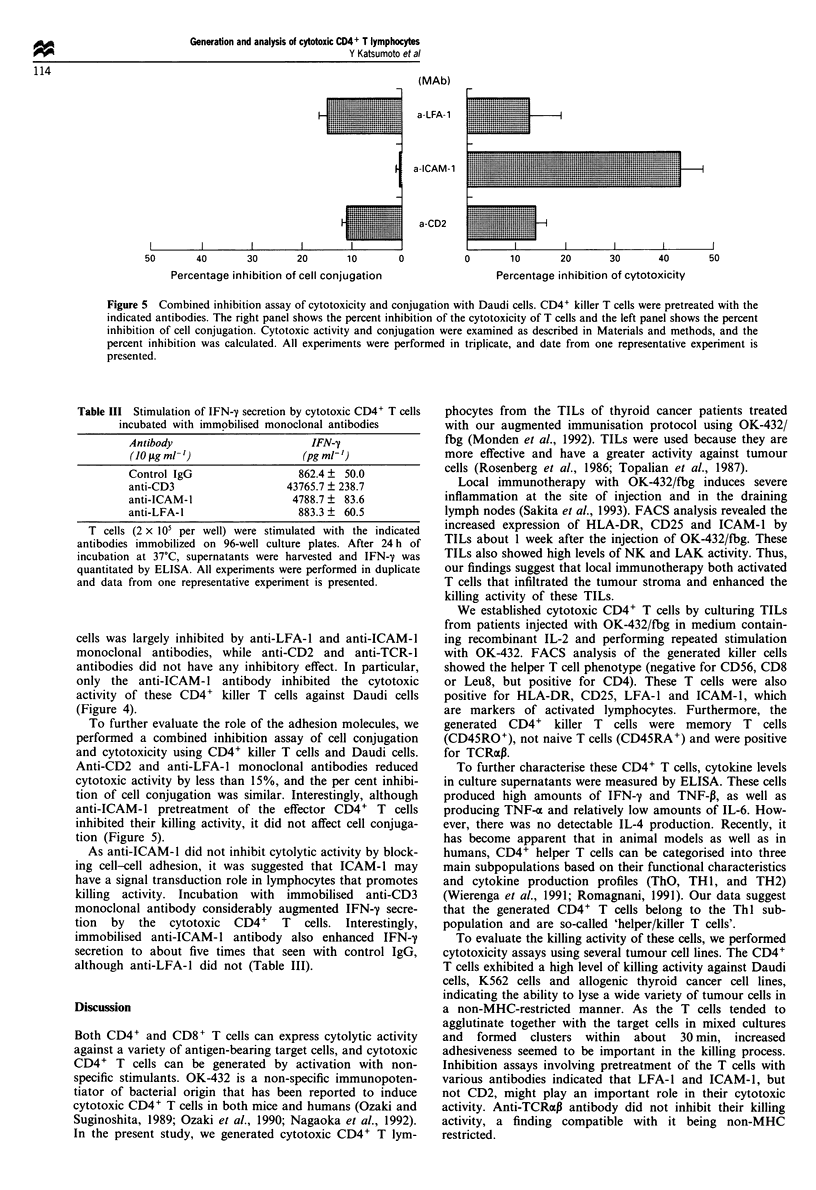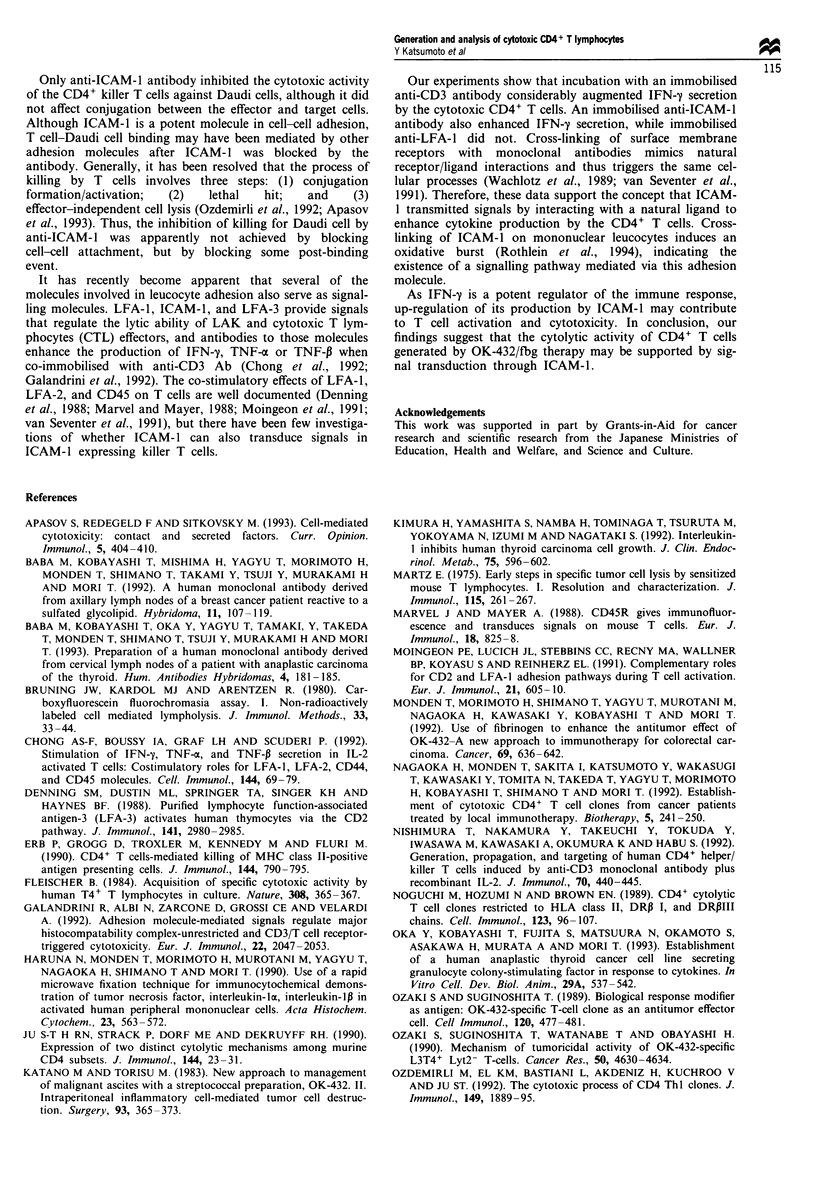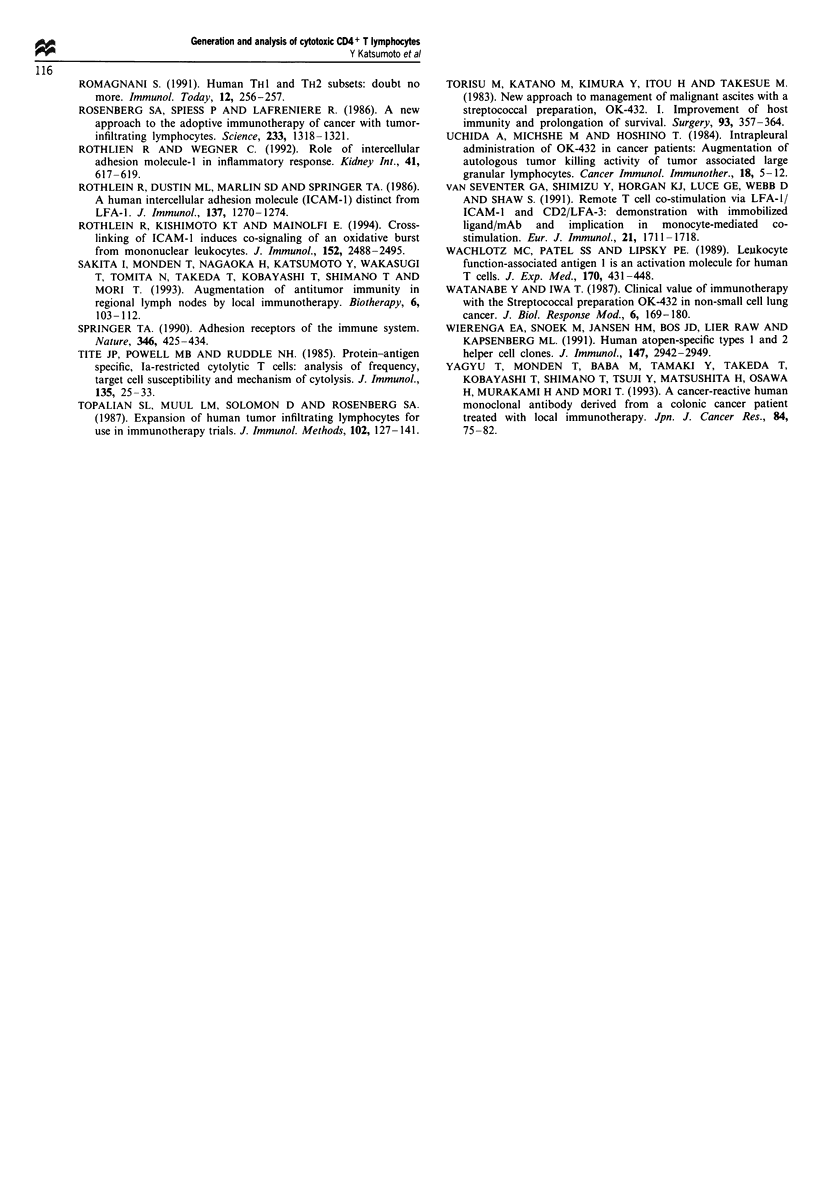# Analysis of cytotoxic activity of the CD4+ T lymphocytes generated by local immunotherapy.

**DOI:** 10.1038/bjc.1996.20

**Published:** 1996-01

**Authors:** Y. Katsumoto, T. Monden, T. Takeda, A. Haba, Y. Ito, E. Wakasugi, T. Wakasugi, M. Sekimoto, T. Kobayashi, H. Shiozaki, T. Shimano, M. Monden

**Affiliations:** Department of Surgery II, Osaka University Medical School, Japan.

## Abstract

We previously reported that the anti-tumour effect of OK-432 is considerably enhanced by its intratumoral injection together with fibrinogen. In the present study, we generated killer T cells by culturing tumour-infiltrating lymphocytes from thyroid cancer patients who had received this local immunotherapy. Phenotypic analysis revealed that the T cells were positive for CD3+, CD4+, Leu8-, CD45RO+ and T-cell receptor (TCR)alpha beta+, as well as showing strong surface expression of HLA-DR, CD25, LFA-1 and ICAM-1. The generated CD4+ T cells secreted interferon (IFN)-gamma, tumour necrosis factor (TNF)-alpha, TNF-beta, and interleukin (IL)-6 (but not IL-4), and exhibited a high level of cytolytic activity against several tumour cell lines. The cytolytic activity of these T cells for Daudi cells was inhibited by preincubation with an anti-intercellular adhesion molecule (ICAM)-1 antibody, but not by preincubation with anti-TCR alpha beta, anti-CD2, or anti-LFA-1 antibodies. Pretreatment with anti-ICAM-1 antibody inhibited T-cell cytolytic activity, but not conjugation with target cells. In addition, incubation with immobilised anti-ICAM-1 enhanced the secretion of IFN-gamma by T cells. We conclude that ICAM-1 expressed on the effector cytotoxic CD4+ T lymphocytes delivers regulatory signals that enhance IFN-gamma secretion.


British Journal of Cancer (1996) 73, 110–116
© 1996 Stockton Press All rights reserved 0007-0920/96 $12.00

### Analysis of cytotoxic activity of the CD4$^+$ T lymphocytes generated by local immunotherapy

Y Katsumoto, T Monden, T Takeda, A Haba, Y Ito, E Wakasugi, T Wakasugi, M Sekimoto, T Kobayashi, H Shiozaki, T Shimano and M Monden

*Department of Surgery II, Osaka University Medical School, Yamada-oka 2-2, Suita, Osaka, 565, Japan.*

**Summary** We previously reported that the anti-tumour effect of OK-432 is considerably enhanced by its intratumoral injection together with fibrinogen. In the present study, we generated killer T cells by culturing tumour-infiltrating lymphocytes from thyroid cancer patients who had received this local immunotherapy. Phenotypic analysis revealed that the T cells were positive for CD3$^+$, CD4$^+$, Leu8$^-$, CD45RO$^+$ and T-cell receptor (TCR)$\alpha\beta^+$, as well as showing strong surface expression of HLA-DR, CD25, LFA-1 and ICAM-1. The generated CD4$^+$ T cells secreted interferon (IFN)-$\gamma$, tumour necrosis factor (TNF)-$\alpha$, TNF-$\beta$, and interleukin (IL)-6 (but not IL-4), and exhibited a high level of cytolytic activity against several tumour cell lines. The cytolytic activity of these T cells for Daudi cells was inhibited by preincubation with an anti-intercellular adhesion molecule (ICAM)-1 antibody, but not by preincubation with anti-TCR$\alpha\beta$. anti-CD2, or anti-LFA-1 antibodies. Pretreatment with anti-ICAM-1 antibody inhibited T-cell cytolytic activity, but not conjugation with target cells. In addition, incubation with immobilised anti-ICAM-1 enhanced the secretion of IFN-$\gamma$ by T cells. We conclude that ICAM-1 expressed on the effector cytotoxic CD4$^+$ T lymphocytes delivers regulatory signals that enhance IFN-$\gamma$ secretion.

**Keywords:** cytotoxic CD4$^+$ T cell; fibrinogen; immunotherapy; ICAM-1; OK-432; thyroid cancer

The streptococcal preparation OK-432 is an immunostimulator produced by treating the Su strain of *Streptococcus pyogenes* with heat and penicillin, and it has been recognised as one of the most effective biological response modifiers (BRMs) for augmenting host immunity (Katano and Torisu, 1983; Torisu *et al*., 1983; Uchida *et al*., 1984; Watanabe & Iwa, 1987; Haruna *et al*., 1990). We have previously shown that the anti-tumour activity of OK-432 is considerably enhanced by intratumoral injection together with fibrinogen (Monden *et al*., 1992). This local immunotherapy effectively induces the marked infiltration of inflammatory cells into the tumour stroma on the day after injection and can contribute to tumour regression. We have also demonstrated that the regional lymph nodes of cancer patients receiving this local immunotherapy were a useful source of competent B lymphocytes for the establishment of human–human hybridomas (Baba *et al*., 1992; Yagyu *et al*., 1993), and we have established cytotoxic CD4$^+$ T-cell clones from such lymph nodes (Nagaoka *et al*., 1992). In the present study, we generated cytotoxic CD4$^+$ T cells from the tumour-infiltrating lymphocytes (TILs) of thyroid cancer patients who had received the intratumoral injection of OK-432 with fibrinogen.

CD4 and CD8 are cell-surface molecules that are expressed on mutually exclusive subsets of T lymphocytes. CD8$^+$ T lymphocytes mediate cytotoxic activity against target cells that are recognised in an MHC class I-restricted fashion.

In contrast, the major function of CD4$^+$ T cells, which recognise antigens that bind to MHC class II molecules, is to promote antibody production by B cells and the secretion of a number of biologically important cytokines. Several reports have documented that both CD4$^+$ and CD8$^+$ cells show cytolytic activity when activated by target cells bearing the appropriate antigen or by non-specific stimulants (Fleischer, 1984; Tite *et al*., 1985; Noguchi *et al*., 1989; Erb *et al*., 1990; Ju *et al*., 1990). Cytolysis mediated by CD8$^+$ T lymphocytes is now well documented, but as is the case for CD4$^+$ cells, the mechanisms involved are still controversial (Nagaoka *et al*., 1992; Nishimura *et al*., 1992; Ozdemirli *et al*., 1992; Apasov *et al*., 1993).

The adhesion molecules expressed on T lymphocytes are crucial for stabilising cell–cell interactions. They were first thought to be accessory molecules that simply joined one cell to another. Recently, it has been realised that these molecules also transmit signals from outside the cell to the inside (Wachlotz *et al*., 1989; van Seventer *et al*., 1991; Galandrini *et al*., 1992). Intercellular adhesion molecule-1 (ICAM-1) is a major ligand for LFA-1 (CD11a/CD18), Mac-1 (CD11b/CD18), and the major group of rhinoviruses. It is expressed on a variety of cells in inflammatory lesions and is induced *in vivo* and *in vitro* by cytokines such as interleukin(IL)-1, tumour necrosis factor (TNF)-$\alpha$, or interferon (IFN)-$\gamma$ (Springer, 1990; Rothlein *et al*., 1986; Rothlein and Wegner, 1992).

In this study, we analysed the characteristics and the killing mechanisms of cytotoxic CD4$^+$ T lymphocytes generated by local immunotherapy, as well as the role of ICAM-1 expression of these T cells in the cytotoxic activity of these cells.

Correspondence: Y Katsumoto
Received 26 June 1995; accepted 28 July 1995

### Materials and methods

### *Local immunotherapy*

A solution for immumotherapy was prepared by dissolving 5 Klinische Einheit (KE; 1 KE corresponds to 0.1 mg of lyophilised *Streptococcus pyogenes*) of OK-432 (Chugai Pharmaceutical, Tokyo, Japan), in 1 ml of aprotinin (1000 Kallikrein Inhibitor Einheit; KIE), and adding 80 mg of human heat-inactivated fibrinogen (Beriplast P; Behring-Werke, Marburg, Germany) containing blood coagulation factor XIII to produce OK-432/fibrinogen solution (OK-432/fbg). At 4–7 days before surgery, seven patients with thyroid cancer received the intratumoral injection of this solution.

### *Preparation of TILs from surgical specimens*

Under sterile condition, surgically resected tumour specimens were immediately minced with scissors and incubated for 2 h at 37°C in AIM-V medium (Gibco, NY, USA) containing type IV collagenase (200 U ml$^{-1}$, Sigma, MO, USA), hyaluronidase (30 U ml$^{-1}$, Sigma), and deoxyribonuclease (100 $\mu$g ml$^{-1}$, Sigma). After passage through a nylon mesh to remove undigested tissue fragments, the cells were centrifuged on lymphocyte separation medium (LSM; Organon Tecknica, NC, USA). Then the TILs at the interface were collected, washed and resuspended in the same medium.

*Generation of cytotoxic T lymphocytes*

TILs from patients pretreated with OK-432/fbg were cultured in AIM-V medium containing 1000 U $ml^{-1}$ of recombinant IL-2 (Shionogi Pharmaceutical, Osaka, Japan). Cells adherent to the plastic plate were removed and non-adherent cells were stimulated biweekly with 0.05 KE $ml^{-1}$ of OK-432. This resulted in the selective growth of $CD4^+$ T lymphocytes after 4 weeks of incubation. As a control, TILs were harvested from patients without local immunotherapy and cultured under the same conditions.

*Flow cytometric analysis*

Flow cytometric analysis of lymphocyte surface phenotypes was carried out by direct immunofluorescence using a FACScan (Becton Dickinson, Mountain View, CA, USA). Lymphocytes were incubated for 30 min at 4°C with 20 $\mu$l of the appropriate dilution of the following fluorescein isothiocyanate (FITC)-conjugated or phycoerythrin (PE)-conjugated monoclonal antibodies: anti-Leu 4 (CD3), anti-Leu 2a (CD8), anti-Leu 3a (CD4), anti-Leu 8, anti-CD2 (LFA-2), anti-HLA-DR for HLA Class II, anti-CD25 for p55-IL-2 receptor, anti-TCR-1 (TCR$\alpha\beta$), anti-Leu45RO (CD45RO), anti-Leu 18 (CD45RA) (Becton Dickinson), anti-CD11a (LFA-1$\alpha$ chain) and anti-CD54 (ICAM-1) (Immunotech, Marseille, France).

*Cytokine assay*

The generated $CD4^+$ T lymphocytes ($1 \times 10^7$) were cultured in AIM-V medium containing 1000 U $ml^{-1}$ of recombinant IL-2 and 0.05 KE $ml^{-1}$ of OK-432 in a total volume of 10 ml. After 48 h, the supernatants were used for the assay of cytokines.

In some experiments, $CD4^+$ T cells were stimulated with immobilised monoclonal antibodies. A 96-well, flat-bottomed cell culture cluster dish (Costar, Cambridge, MA, USA) was coated with 10 $\mu g\ ml^{-1}$ of anti-ICAM-1, anti-LFA-1, or anti-CD3 monoclonal antibodies. Normal mouse serum containing 10 $\mu g\ ml^{-1}$ of non-specific IgG was used as the negative control. After overnight incubation at 4°C, the excess antibodies were removed and the plates were washed twice with phosphate-buffered saline (PBS). Then the plates were used to stimulate $CD4^+$ T lymphocytes by culturing the T cells ($2 \times 10^5$ per well) in a total volume of 200 $\mu$l for 24 h at 37°C. Cell-free supernatants were harvested and stored at −20°C until quantitation of the IFN-$\gamma$ content.

Cytokines were detected by the two-site sandwich enzyme-linked immunosorbent assay (ELISA) technique using monoclonal antibodies to coat the solid phase and as the secondary antibody. The ELISA kits for IFN-$\gamma$ and IL-4 were obtained from Medgenix (Fleurus, Belgium), while that for IL-6 was from Toray Industries (Tokyo, Japan), that for TNF-$\alpha$ came from Otsuka Pharmaceutical (Tokyo, Japan), and that for TNF-$\beta$ was from Bender Depl. MedSystems (Vienna, Austria). Samples were assayed in duplicate and quantitated by comparison with standard curves obtained using purified recombinant or natural cytokines.

*Cytotoxicity assay*

The following target cells were used: K562 (a natural killer (NK)-sensitive myeloid leukemia cell line), Daudi (an NK-resistant Burkitt's lymphoma cell line), KOA-2 and K-119 [anaplastic thyroid carcinoma cell lines established in our laboratory (Baba *et al.*, 1993; Oka *et al.*, 1993)], and NPA [a papillary thyroid carcinoma cell line kindly provided by Professor S Yamashita; Department of Cell Physiology, Atomic Disease Institute, Nagasaki University School of Medicine (Kimura *et al.*, 1992)]. Cytolytic activity was assessed by a carboxyfluorescein fluorochromasia assay (Bruning *et al.*, 1980). Briefly, the target cells were labelled with 5-carboxyfluorescein diacetate (40 $\mu g\ ml^{-1}$, Sigma) for 1 h at 37°C. Effector T cells were then added to target cells ($1 \times 10^4$ per well) in a 60-well Terasaki plate (Greiner, Frickenkaiser, Germany) at various effector–target ratios, and were incubated for 3 h at 37°C.

As a negative control, medium without effector cells was added, and 0.2% Nonidet P-40 (Sigma) was used as the positive control. After incubation, 5 $\mu$l of clarified bovine haemoglobin (5 mM, Sigma) was added to diminish the background fluorescence released from lysed target cells. After measuring the fluorescence intensity of the surviving target cells with an MPV compact MT (Leitz, Wetzlar, Germany), the per cent lysis was calculated by the following formula:

$$\left(1 - \frac{\text{Mean experimental reading} - \text{mean positive control reading}}{\text{Mean negative control reading} - \text{mean positive control reading}}\right) \times 100\ (\%)$$

*Cell conjugation assay*

Conjugation between the $CD4^+$ T cells and target cells was assessed as described previously with slight modifications (Martz, 1975; Ozdemirli *et al.*, 1992).

Effector cells ($5 \times 10^5$) were mixed with target cells ($5 \times 10^4$) in total volume of 100 $\mu$l in 96-well U-bottomed micro plates (Costar, Cambridge, MA, USA). After incubation at 37°C in a humidified carbon dioxide incubator for 3 h, the cells were attached to glass slides using a CF-12D auto smear (Sakura Seiki, Tokyo, Japan) at 800 r.p.m. for 3 min. The cells were then overlaid with 100 $\mu$l of a mixture containing 0.05% glutaraldehyde, 2% formaldehyde, 0.025% calcium chloride and 0.1 M sodium cacodylate in PBS (pH 7.4), and were fixed by a microwave irradiation at 500 W for 10 s in a domestic microwave oven (Haruna *et al.*, 1990). At least 100 target cells were counted under the microscope and the per cent conjugation was calculated as the number of conjugated target cells divided by the total number of target cells. The large target cells were easily distinguished from the small effector cells and conjugation was arbitrarily defined as the binding of at least three effector cells to a target cell. Three different microscopic fields were examined in each experiment.

**Figure 1** Flow cytometric analysis of tumor-infiltrating lymphocytes using anti-HLA-DR, anti-CD25, anti-ICAM-1, and anti-CD3 antibodies (a), and cytotoxicity assay using Daudi and K562 cells (b). Lymphocytes from patients pretreated with OK-432/fibrinogen (▩) are compared with those from patients without immunotherapy (□). *: $p<0.05$; **: $p<0.01$.

**Table I** Phenotypic changes and cytotoxic activity of TILs during culture

| | *Weeks of culture* | *Surface expression (%)* *CD4/T* | *CD8/T* | *HLA-DR/T* | *CD25/T* | *ICAM-1/T* | *Cytotoxicity (%)* *K562* | *Daudi* |
|---|---|---|---|---|---|---|---|---|
| *OK-432/fbg treated* | | | | | | | | |
| Case 1 | 0 | 74.8 | 25.2 | 59.9 | 15.9 | 33.0 | 69.2 | 55.0 |
| | 2 | 96.4 | 3.6 | 86.9 | 79.4 | 84.6 | 57.9 | 56.6 |
| | 4 | 95.0 | 5.0 | 95.6 | 53.7 | 86.9 | 60.0 | 54.0 |
| Case 2 | 0 | – | – | – | – | – | – | – |
| | 2 | 83.3 | 16.7 | 92.8 | 77.6 | 77.9 | 52.0 | 89.0 |
| | 4 | 98.0 | 2.0 | 91.6 | 76.8 | 67.1 | 29.0 | 65.0 |
| Case 3 | 0 | 69.6 | 30.4 | 70.8 | 10.4 | 39.3 | – | – |
| | 2 | 56.0 | 44.0 | 86.5 | 34.2 | 76.0 | – | – |
| | 4 | 99.8 | 0.2 | 86.0 | 67.0 | 97.0 | 63.4 | 72.7 |
| Case 4 | 0 | 65.0 | 35.0 | 52.2 | 27.4 | 72.6 | – | – |
| | 2 | 84.8 | 15.2 | 91.4 | 65.7 | 97.9 | – | 21.3 |
| | 4 | 96.3 | 3.7 | 93.6 | 54.8 | 66.7 | – | 56.1 |
| Case 5 | 0 | 67.1 | 32.9 | 71.6 | 28.0 | 39.2 | – | – |
| | 2 | 88.1 | 11.9 | – | – | – | – | – |
| | 4 | 91.8 | 8.2 | 99.3 | 81.9 | 67.5 | 63.4 | 60.7 |
| Case 6 | 0 | 84.7 | 15.3 | 71.1 | 13.9 | 35.1 | – | – |
| | 2 | 72.5 | 27.5 | – | – | 57.9 | – | – |
| | 4 | 97.5 | 2.5 | 97.3 | 86.6 | 95.9 | 75.8 | 51.4 |
| Case 7 | 0 | 72.8 | 27.2 | 53.8 | 9.8 | 66.5 | 23.9 | 53.0 |
| | 2 | 95.9 | 4.1 | 84.0 | 45.0 | 99.5 | 47.7 | 53.6 |
| | 4 | 99.1 | 0.9 | 99.3 | 55.7 | 77.9 | 35.2 | 57.8 |
| *Control* | | | | | | | | |
| Case 1 | 0 | 55.6 | 44.4 | 28.4 | 13.1 | 19.9 | 0.0 | 0.0 |
| | 2 | 16.1 | 83.9 | 97.8 | 11.5 | 49.5 | 2.0 | 0.0 |
| | 4 | 1.9 | 98.1 | 93.0 | 18.5 | 41.2 | 50.6 | 53.2 |
| Case 2 | 0 | 68.3 | 31.7 | 38.9 | 7.2 | 4.3 | 19.8 | 9.3 |
| | 2 | 59.3 | 40.7 | 70.7 | 12.7 | 33.2 | 3.6 | 10.0 |
| | 4 | 7.4 | 92.6 | 84.8 | 9.6 | 51.5 | 47.6 | 47.6 |
| Case 3 | 0 | 75.1 | 24.9 | 37.7 | 3.4 | 11.6 | 12.2 | 0.0 |
| | 2 | – | – | – | – | – | – | – |
| | 4 | – | – | – | – | – | – | – |
| Case 4 | 0 | 62.4 | 37.6 | 51.6 | 6.9 | 37.7 | – | – |
| | 2 | – | – | – | – | – | – | – |
| | 4 | – | – | – | – | – | – | – |

TILs from patients pretreated with OK-432/fbg (upper panel) and from patients without immunotherapy (lower panel) were cultured with IL-2 and OK-432, after which phenotypic analysis and the cytotoxicity assay were performed.

**Figure 2** Phenotype of cytotoxic CD4+ T lymphocytes. T cells were stained with phycoerythrin-conjugated and FITC-conjugated antibodies for analysis by two-color flow cytometry. Data from representative experiments are expressed as contour plots of FITC and phycoerythrin fluorescence on 4-decade log scales. Quadrant markers were positioned to include >98% of control cells in the lower left quadrant. The percentage of cells within each quadrant is indicated.

*Inhibition assay*

Cytotoxic T lymphocytes were preincubated with anti-ICAM-1, anti-LFA-1, anti-CD2 and anti-TCR-1 monoclonal antibodies (2 $\mu$g ml$^{-1}$) for 1 h at 37°C. After washing twice in AIM-V to remove excess antibodies these effector cells were used for the cytotoxicity and conjugation assays as described above.

### Results

We analysed TILs from seven thyroid cancer patients who received intratumoral injection of OK-432/fbg, as well as cells from four patients without immunotherapy. FACS analysis of TILs from the former patients showed increased surface expression of HLA-DR, CD25, and ICAM-1 when compared with cells from the latter patients (Figure 1).

In the OK-432/fbg group, phenotypic analysis of lymphocytes cultured in medium containing recombinant IL-2 revealed that CD4$^+$ T cells gradually increased after repeated stimulation with OK-432, and accounted for >90% of all cells after several weeks. In contrast, CD8$^+$ T lymphocytes were dominant among cells from the patients without immunotherapy after the same duration of culture. Following culture of cells from the OK-432/fbg group with repeated stimulation for 4 weeks, CD8$^+$ T cells virtually disappeared and CD4$^+$ T cells reached almost 100% (Table I).

The *in vitro* cytotoxicity assay showed that local immunotherapy considerably enhanced NK and lymphokine activated killer (LAK) activity of TILs (Figure 1). During culture, the cytolytic activity of TILs from the OK-432/fbg group was not reduced despite the expansion of CD4$^+$ T cells, although TILs from the control group only showed cytolytic activity when CD8$^+$-LAK cells were generated (Table I). Figure 2 shows the representative phenotype of cytotoxic CD4$^+$ T cells from seven patients after culture for more than 5 weeks. FACS analysis revealed that the proliferating T cells expressed the helper T phenotype (CD4$^+$ and Leu8$^-$). The majority of the T cells were positive for T cell receptor (TCR) $\alpha\beta$, CD45RO and LFA-1, and the surface expression of HLA-DR, the IL-2 receptor (CD25), and ICAM-1 gradually increased throughout culture (Table I).

To further characterise these CD4$^+$ T cells, cytokine production in culture supernatants was measured by ELISA. CD4$^+$ T cells stimulated with OK-432 produced high amounts of IFN-$\gamma$ and TNF-$\beta$, but no IL-4 was secreted by these T cells. Other cytokines, such as TNF-$\alpha$ and IL-6, were also detected in the culture supernatants. The results are summarised in Table II.

The cytotoxicity assay revealed that these T cells exhibited a high level of killing activity against a broad spectrum of target cells, including Daudi cells, K562 cells and allogenic thyroid cancer cell lines (NPA, KOA-2 and K-119), but did not kill autologous or allogenic peripheral blood lymphocytes (Figure 3). Once activated, the cytotoxic CD4$^+$ T cells did not require OK-432 to express their cytotoxicity, because addition of OK-432 to the assay system at various concentrations did not alter the cytolysis of Daudi or K562 cells (data not shown).

This suggested that the killing activity of these CD4$^+$ T cells was non-MHC-restricted and that their cytotoxic activity might be supported by other surface molecules such as adhesion molecules. To evaluate the role of such molecules in cytotoxicity we performed an inhibition assay. The cytotoxicity of CD4$^+$ killer T cells against KOA-2 and K-119

**Table II** Cytokine secretion by cytotoxic CD4$^+$ T cells (pg ml$^{-1}$)

| | |
|---|---|
| IFN-$\gamma$ | 46250.0 ± 2450.0 |
| TNF-$\beta$ | 5285.0 ± 295.0 |
| TNF-$\alpha$ | 343.2 ± 64.3 |
| IL-4 | <6.0 ± 0.0 |
| IL-6 | 121.6 ± 14.2 |

T cells (1 × 10$^7$) were cultured for 48 h at 37°C in the presence of IL-2 and OK-432, the supernatants were harvested and were tested for the indicated cytokines by ELISA. All experiments were performed in duplicate and the mean ± s.d. is presented.

**Figure 3** Cytotoxicity of the generated CD8$^+$ killer (■) and CD4$^+$ killer (▒) T cells for K562 cells, Daudi cells, allogenic thyroid cancer cell lines, and peripheral blood lymphocytes (PBL). Cytotoxicity was determined by a 3 h carboxyfluorescein fluorochromasia assay at an effector/target ratio of 10:1. *: Not tested.

**Figure 4** Inhibition of the cytotoxicity of generated CD8$^+$ (■) and CD4$^+$ (▒) killer T cells for various tumour cell lines. Killer T cells were preincubated with the indicated monoclonal antibodies, and the percent inhibition of cytotoxicity for Daudi cells (a), KOA-2 cells (b), and K 119 cells (c) was calculated. All experiments were performed in triplicate, and data from one representative experiment is presented.

**Figure 5** Combined inhibition assay of cytotoxicity and conjugation with Daudi cells. $CD4^+$ killer T cells were pretreated with the indicated antibodies. The right panel shows the percent inhibition of the cytotoxicity of T cells and the left panel shows the percent inhibition of cell conjugation. Cytotoxic activity and conjugation were examined as described in Materials and methods, and the percent inhibition was calculated. All experiments were performed in triplicate, and date from one representative experiment is presented.

**Table III** Stimulation of IFN-γ secretion by cytotoxic $CD4^+$ T cells incubated with immobilised monoclonal antibodies

| *Antibody (10 μg ml⁻¹)* | *IFN-γ (pg ml⁻¹)* |
|---|---|
| Control IgG | 862.4 ± 50.0 |
| anti-CD3 | 43765.7 ± 238.7 |
| anti-ICAM-1 | 4788.7 ± 83.6 |
| anti-LFA-1 | 883.3 ± 60.5 |

T cells ($2 \times 10^5$ per well) were stimulated with the indicated antibodies immobilized on 96-well culture plates. After 24 h of incubation at 37°C, supernatants were harvested and IFN-γ was quantitated by ELISA. All experiments were performed in duplicate and data from one representative experiment is presented.

cells was largely inhibited by anti-LFA-1 and anti-ICAM-1 monoclonal antibodies, while anti-CD2 and anti-TCR-1 antibodies did not have any inhibitory effect. In particular, only the anti-ICAM-1 antibody inhibited the cytotoxic activity of these $CD4^+$ killer T cells against Daudi cells (Figure 4).

To further evaluate the role of the adhesion molecules, we performed a combined inhibition assay of cell conjugation and cytotoxicity using $CD4^+$ killer T cells and Daudi cells. Anti-CD2 and anti-LFA-1 monoclonal antibodies reduced cytotoxic activity by less than 15%, and the per cent inhibition of cell conjugation was similar. Interestingly, although anti-ICAM-1 pretreatment of the effector $CD4^+$ T cells inhibited their killing activity, it did not affect cell conjugation (Figure 5).

As anti-ICAM-1 did not inhibit cytolytic activity by blocking cell–cell adhesion, it was suggested that ICAM-1 may have a signal transduction role in lymphocytes that promotes killing activity. Incubation with immobilised anti-CD3 monoclonal antibody considerably augmented IFN-γ secretion by the cytotoxic $CD4^+$ T cells. Interestingly, immobilised anti-ICAM-1 antibody also enhanced IFN-γ secretion to about five times that seen with control IgG, although anti-LFA-1 did not (Table III).

### Discussion

Both $CD4^+$ and $CD8^+$ T cells can express cytolytic activity against a variety of antigen-bearing target cells, and cytotoxic $CD4^+$ T cells can be generated by activation with non-specific stimulants. OK-432 is a non-specific immunopotentiator of bacterial origin that has been reported to induce cytotoxic $CD4^+$ T cells in both mice and humans (Ozaki and Suginoshita, 1989; Ozaki *et al.*, 1990; Nagaoka *et al.*, 1992). In the present study, we generated cytotoxic $CD4^+$ T lymphocytes from the TILs of thyroid cancer patients treated with our augmented immunisation protocol using OK-432/fbg (Monden *et al.*, 1992). TILs were used because they are more effective and have a greater activity against tumour cells (Rosenberg *et al.*, 1986; Topalian *et al.*, 1987).

Local immunotherapy with OK-432/fbg induces severe inflammation at the site of injection and in the draining lymph nodes (Sakita *et al.*, 1993). FACS analysis revealed the increased expression of HLA-DR, CD25 and ICAM-1 by TILs about 1 week after the injection of OK-432/fbg. These TILs also showed high levels of NK and LAK activity. Thus, our findings suggest that local immunotherapy both activated T cells that infiltrated the tumour stroma and enhanced the killing activity of these TILs.

We established cytotoxic $CD4^+$ T cells by culturing TILs from patients injected with OK-432/fbg in medium containing recombinant IL-2 and performing repeated stimulation with OK-432. FACS analysis of the generated killer cells showed the helper T cell phenotype (negative for CD56, CD8 or Leu8, but positive for CD4). These T cells were also positive for HLA-DR, CD25, LFA-1 and ICAM-1, which are markers of activated lymphocytes. Furthermore, the generated $CD4^+$ killer T cells were memory T cells ($CD45RO^+$), not naive T cells ($CD45RA^+$) and were positive for TCRαβ.

To further characterise these $CD4^+$ T cells, cytokine levels in culture supernatants were measured by ELISA. These cells produced high amounts of IFN-γ and TNF-β, as well as producing TNF-α and relatively low amounts of IL-6. However, there was no detectable IL-4 production. Recently, it has become apparent that in animal models as well as in humans, $CD4^+$ helper T cells can be categorised into three main subpopulations based on their functional characteristics and cytokine production profiles (ThO, TH1, and TH2) (Wierenga *et al.*, 1991; Romagnani, 1991). Our data suggest that the generated $CD4^+$ T cells belong to the Th1 subpopulation and are so-called 'helper/killer T cells'.

To evaluate the killing activity of these cells, we performed cytotoxicity assays using several tumour cell lines. The $CD4^+$ T cells exhibited a high level of killing activity against Daudi cells, K562 cells and allogenic thyroid cancer cell lines, indicating the ability to lyse a wide variety of tumour cells in a non-MHC-restricted manner. As the T cells tended to agglutinate together with the target cells in mixed cultures and formed clusters within about 30 min, increased adhesiveness seemed to be important in the killing process. Inhibition assays involving pretreatment of the T cells with various antibodies indicated that LFA-1 and ICAM-1, but not CD2, might play an important role in their cytotoxic activity. Anti-TCRαβ antibody did not inhibit their killing activity, a finding compatible with it being non-MHC restricted.

Only anti-ICAM-1 antibody inhibited the cytotoxic activity of the CD4+ killer T cells against Daudi cells, although it did not affect conjugation between the effector and target cells. Although ICAM-1 is a potent molecule in cell–cell adhesion, T cell–Daudi cell binding may have been mediated by other adhesion molecules after ICAM-1 was blocked by the antibody. Generally, it has been resolved that the process of killing by T cells involves three steps: (1) conjugation formation/activation; (2) lethal hit; and (3) effector–independent cell lysis (Ozdemirli *et al.*, 1992; Apasov *et al.*, 1993). Thus, the inhibition of killing for Daudi cell by anti-ICAM-1 was apparently not achieved by blocking cell–cell attachment, but by blocking some post-binding event.

It has recently become apparent that several of the molecules involved in leucocyte adhesion also serve as signalling molecules. LFA-1, ICAM-1, and LFA-3 provide signals that regulate the lytic ability of LAK and cytotoxic T lymphocytes (CTL) effectors, and antibodies to those molecules enhance the production of IFN-γ, TNF-α or TNF-β when co-immobilised with anti-CD3 Ab (Chong *et al.*, 1992; Galandrini *et al.*, 1992). The co-stimulatory effects of LFA-1, LFA-2, and CD45 on T cells are well documented (Denning *et al.*, 1988; Marvel and Mayer, 1988; Moingeon *et al.*, 1991; van Seventer *et al.*, 1991), but there have been few investigations of whether ICAM-1 can also transduce signals in ICAM-1 expressing killer T cells.

Our experiments show that incubation with an immobilised anti-CD3 antibody considerably augmented IFN-γ secretion by the cytotoxic CD4+ T cells. An immobilised anti-ICAM-1 antibody also enhanced IFN-γ secretion, while immobilised anti-LFA-1 did not. Cross-linking of surface membrane receptors with monoclonal antibodies mimics natural receptor/ligand interactions and thus triggers the same cellular processes (Wachlotz *et al.*, 1989; van Seventer *et al.*, 1991). Therefore, these data support the concept that ICAM-1 transmitted signals by interacting with a natural ligand to enhance cytokine production by the CD4+ T cells. Cross-linking of ICAM-1 on mononuclear leucocytes induces an oxidative burst (Rothlein *et al.*, 1994), indicating the existence of a signalling pathway mediated via this adhesion molecule.

As IFN-γ is a potent regulator of the immune response, up-regulation of its production by ICAM-1 may contribute to T cell activation and cytotoxicity. In conclusion, our findings suggest that the cytolytic activity of CD4+ T cells generated by OK-432/fbg therapy may be supported by signal transduction through ICAM-1.

**Acknowledgements**
This work was supported in part by Grants-in-Aid for cancer research and scientific research from the Japanese Ministries of Education, Health and Welfare, and Science and Culture.

**References**

APASOV S, REDEGELD F AND SITKOVSKY M. (1993). Cell-mediated cytotoxicity: contact and secreted factors. *Curr. Opinion. Immunol.*, **5**, 404–410.

BABA M, KOBAYASHI T, MISHIMA H, YAGYU T, MORIMOTO H, MONDEN T, SHIMANO T, TAKAMI Y, TSUJI Y, MURAKAMI H AND MORI T. (1992). A human monoclonal antibody derived from axillary lymph nodes of a breast cancer patient reactive to a sulfated glycolipid. *Hybridoma*, **11**, 107–119.

BABA M, KOBAYASHI T, OKA Y, YAGYU T, TAMAKI, Y, TAKEDA T, MONDEN T, SHIMANO T, TSUJI Y, MURAKAMI H AND MORI T. (1993). Preparation of a human monoclonal antibody derived from cervical lymph nodes of a patient with anaplastic carcinoma of the thyroid. *Hum. Antibodies Hybridomas*, **4**, 181–185.

BRUNING JW, KARDOL MJ AND ARENTZEN R. (1980). Carboxyfluorescein fluorochromasia assay. I. Non-radioactively labeled cell mediated lympholysis. *J. Immunol. Methods.*, **33**, 33–44.

CHONG AS-F, BOUSSY IA, GRAF LH AND SCUDERI P. (1992). Stimulation of IFN-γ, TNF-α, and TNF-β secretion in IL-2 activated T cells: Costimulatory roles for LFA-1, LFA-2, CD44, and CD45 molecules. *Cell. Immunol.*, **144**, 69–79.

DENNING SM, DUSTIN ML, SPRINGER TA, SINGER KH AND HAYNES BF. (1988). Purified lymphocyte function-associated antigen-3 (LFA-3) activates human thymocytes via the CD2 pathway. *J. Immunol.*, **141**, 2980–2985.

ERB P, GROGG D, TROXLER M, KENNEDY M AND FLURI M. (1990). CD4+ T cells-mediated killing of MHC class II-positive antigen presenting cells. *J. Immunol.*, **144**, 790–795.

FLEISCHER B. (1984). Acquisition of specific cytotoxic activity by human T4+ T lymphocytes in culture. *Nature*, **308**, 365–367.

GALANDRINI R, ALBI N, ZARCONE D, GROSSI CE AND VELARDI A. (1992). Adhesion molecule-mediated signals regulate major histocompatability complex-unrestricted and CD3/T cell receptor-triggered cytotoxicity. *Eur. J. Immunol.*, **22**, 2047–2053.

HARUNA N, MONDEN T, MORIMOTO H, MUROTANI M, YAGYU T, NAGAOKA H, SHIMANO T AND MORI T. (1990). Use of a rapid microwave fixation technique for immunocytochemical demonstration of tumor necrosis factor, interleukin-1α, interleukin-1β in activated human peripheral mononuclear cells. *Acta Histochem. Cytochem.*, **23**, 563–572.

JU S-T H RN, STRACK P, DORF ME AND DEKRUYFF RH. (1990). Expression of two distinct cytolytic mechanisms among murine CD4 subsets. *J. Immunol.*, **144**, 23–31.

KATANO M AND TORISU M. (1983). New approach to management of malignant ascites with a streptococcal preparation, OK-432. II. Intraperitoneal inflammatory cell-mediated tumor cell destruction. *Surgery*, **93**, 365–373.

KIMURA H, YAMASHITA S, NAMBA H, TOMINAGA T, TSURUTA M, YOKOYAMA N, IZUMI M AND NAGATAKI S. (1992). Interleukin-1 inhibits human thyroid carcinoma cell growth. *J. Clin. Endocrinol. Metab.*, **75**, 596–602.

MARTZ E. (1975). Early steps in specific tumor cell lysis by sensitized mouse T lymphocytes. I. Resolution and characterization. *J. Immunol.*, **115**, 261–267.

MARVEL J AND MAYER A. (1988). CD45R gives immunofluorescence and transduces signals on mouse T cells. *Eur. J. Immunol.*, **18**, 825–8.

MOINGEON PE, LUCICH JL, STEBBINS CC, RECNY MA, WALLNER BP, KOYASU S AND REINHERZ EL. (1991). Complementary roles for CD2 and LFA-1 adhesion pathways during T cell activation. *Eur. J. Immunol.*, **21**, 605–10.

MONDEN T, MORIMOTO H, SHIMANO T, YAGYU T, MUROTANI M, NAGAOKA H, KAWASAKI Y, KOBAYASHI T AND MORI T. (1992). Use of fibrinogen to enhance the antitumor effect of OK-432–A new approach to immunotherapy for colorectal carcinoma. *Cancer*, **69**, 636–642.

NAGAOKA H, MONDEN T, SAKITA I, KATSUMOTO Y, WAKASUGI T, KAWASAKI Y, TOMITA N, TAKEDA T, YAGYU T, MORIMOTO H, KOBAYASHI T, SHIMANO T AND MORI T. (1992). Establishment of cytotoxic CD4+ T cell clones from cancer patients treated by local immunotherapy. *Biotherapy*, **5**, 241–250.

NISHIMURA T, NAKAMURA Y, TAKEUCHI Y, TOKUDA Y, IWASAWA M, KAWASAKI A, OKUMURA K AND HABU S. (1992). Generation, propagation, and targeting of human CD4+ helper/killer T cells induced by anti-CD3 monoclonal antibody plus recombinant IL-2. *J. Immunol.*, **70**, 440–445.

NOGUCHI M, HOZUMI N AND BROWN EN. (1989). CD4+ cytolytic T cell clones restricted to HLA class II, DRβ I, and DRβIII chains. *Cell. Immunol.*, **123**, 96–107.

OKA Y, KOBAYASHI T, FUJITA S, MATSUURA N, OKAMOTO S, ASAKAWA H, MURATA A AND MORI T. (1993). Establishment of a human anaplastic thyroid cancer cell line secreting granulocyte colony-stimulating factor in response to cytokines. *In Vitro Cell. Dev. Biol. Anim.*, **29A**, 537–542.

OZAKI S AND SUGINOSHITA T. (1989). Biological response modifier as antigen: OK-432-specific T-cell clone as an antitumor effector cell. *Cell Immunol.*, **120**, 477–481.

OZAKI S, SUGINOSHITA T, WATANABE T AND OBAYASHI H. (1990). Mechanism of tumoricidal activity of OK-432-specific L3T4+ Lyt2− T-cells. *Cancer Res.*, **50**, 4630–4634.

OZDEMIRLI M, EL KM, BASTIANI L, AKDENIZ H, KUCHROO V AND JU ST. (1992). The cytotoxic process of CD4 Th1 clones. *J. Immunol.*, **149**, 1889–95.

ROMAGNANI S. (1991). Human TH1 and TH2 subsets: doubt no more. *Immunol. Today*, **12**, 256–257.

ROSENBERG SA, SPIESS P AND LAFRENIERE R. (1986). A new approach to the adoptive immunotherapy of cancer with tumor-infiltrating lymphocytes. *Science*, **233**, 1318–1321.

ROTHLIEN R AND WEGNER C. (1992). Role of intercellular adhesion molecule-1 in inflammatory response. *Kidney Int.*, **41**, 617–619.

ROTHLEIN R, DUSTIN ML, MARLIN SD AND SPRINGER TA. (1986). A human intercellular adhesion molecule (ICAM-1) distinct from LFA-1. *J. Immunol.*, **137**, 1270–1274.

ROTHLEIN R, KISHIMOTO KT AND MAINOLFI E. (1994). Cross-linking of ICAM-1 induces co-signaling of an oxidative burst from mononuclear leukocytes. *J. Immunol.*, **152**, 2488–2495.

SAKITA I, MONDEN T, NAGAOKA H, KATSUMOTO Y, WAKASUGI T, TOMITA N, TAKEDA T, KOBAYASHI T, SHIMANO T AND MORI T. (1993). Augmentation of antitumor immunity in regional lymph nodes by local immunotherapy. *Biotherapy*, **6**, 103–112.

SPRINGER TA. (1990). Adhesion receptors of the immune system. *Nature*, **346**, 425–434.

TITE JP, POWELL MB AND RUDDLE NH. (1985). Protein-antigen specific, Ia-restricted cytolytic T cells: analysis of frequency, target cell susceptibility and mechanism of cytolysis. *J. Immunol.*, **135**, 25–33.

TOPALIAN SL, MUUL LM, SOLOMON D AND ROSENBERG SA. (1987). Expansion of human tumor infiltrating lymphocytes for use in immunotherapy trials. *J. Immunol. Methods*, **102**, 127–141.

TORISU M, KATANO M, KIMURA Y, ITOU H AND TAKESUE M. (1983). New approach to management of malignant ascites with a streptococcal preparation, OK-432. I. Improvement of host immunity and prolongation of survival. *Surgery*, **93**, 357–364.

UCHIDA A, MICHSHE M AND HOSHINO T. (1984). Intrapleural administration of OK-432 in cancer patients: Augmentation of autologous tumor killing activity of tumor associated large granular lymphocytes. *Cancer Immunol. Immunother.*, **18**, 5–12.

VAN SEVENTER GA, SHIMIZU Y, HORGAN KJ, LUCE GE, WEBB D AND SHAW S. (1991). Remote T cell co-stimulation via LFA-1/ICAM-1 and CD2/LFA-3: demonstration with immobilized ligand/mAb and implication in monocyte-mediated co-stimulation. *Eur. J. Immunol.*, **21**, 1711–1718.

WACHLOTZ MC, PATEL SS AND LIPSKY PE. (1989). Leukocyte function-associated antigen 1 is an activation molecule for human T cells. *J. Exp. Med.*, **170**, 431–448.

WATANABE Y AND IWA T. (1987). Clinical value of immunotherapy with the Streptococcal preparation OK-432 in non-small cell lung cancer. *J. Biol. Response Mod.*, **6**, 169–180.

WIERENGA EA, SNOEK M, JANSEN HM, BOS JD, LIER RAW AND KAPSENBERG ML. (1991). Human atopen-specific types 1 and 2 helper cell clones. *J. Immunol.*, **147**, 2942–2949.

YAGYU T, MONDEN T, BABA M, TAMAKI Y, TAKEDA T, KOBAYASHI T, SHIMANO T, TSUJI Y, MATSUSHITA H, OSAWA H, MURAKAMI H AND MORI T. (1993). A cancer-reactive human monoclonal antibody derived from a colonic cancer patient treated with local immunotherapy. *Jpn. J. Cancer Res.*, **84**, 75–82.